# A cross-sectional study to explore postgraduate students’ understanding of and beliefs about sexual and reproductive health in a public university, Malaysia

**DOI:** 10.1186/s12978-015-0070-3

**Published:** 2015-08-29

**Authors:** Shahla Soleymani, Hejar Abdul Rahman, Rampal Lekhraj, Nor Afiah Mohd Zulkefli, Nasrin Matinnia

**Affiliations:** Department of Community Health, Faculty of Medicine and Health Sciences, Universiti Putra Malaysia, 43400 UPM, Serdang, Malaysia; Department of Nursing, College of Science, Hamedan branch, Islamic Azad University Hamedan, Hamedan, Iran

## Abstract

**Background:**

The main sexual and reproductive health issues among young people are premarital sexual intercourse, unwanted pregnancies, unsafe abortions and sexually transmitted diseases including Human Immunodeficiency Virus. The aim of this study was to determine the knowledge related to sexual and reproductive health among Malaysian postgraduate students in a public university in Malaysia.

**Methods:**

A cross-sectional study was carried out among postgraduate students by systematic random sampling technique. A pre-tested self administered questionnaire was used to collect the data.

**Results:**

Out of 434 respondents, the majority of students were female (78.6 %) and single (78.3 %). The overall mean age of respondents was 27.0 ranging from 20 to 46 years of age. The main sources of information for sexual and reproductive health awareness were the internet (78.6 %) and newspaper (61.8 %). The majority (97.9 %) of the students knew that AIDS is a sexually transmitted disease. Most of them believed that the spread of sexually transmitted diseases was through shaking hands (92.1 %). Use of condoms was perceived to be the best way to avoid sexually transmitted diseases (88.4 %). Sexual and reproductive health knowledge was significantly associated with the students’ age, marital status and faculty. The socio-demographic factors and current educational status accounted for a significant 9 % of the variability in sexual and reproductive health knowledge, *f* (7, 426) = 11, *p* <0.001.

**Conclusions:**

The postgraduate students’ level of knowledge on sexual and reproductive health was not satisfactory. Sexual and reproductive health knowledge was associated with the students’ marital status and faculty. Intervention programs related to sexual and reproductive health are recommended.

## Background

Sexual and reproductive health (SRH) is an essential part of public health and can considerably influence the general well being and quality of life [1]. Reproductive health includes all issues related to the reproductive system and its function while sexual health indicates the ability of having a safe and satisfying sex life [2].

Annually about 500 million new cases of sexually transmitted diseases (STD) are reported all around the world among people aged 15–49 years old excluding human immunodeficiency virus (HIV) and other sexually transmitted infections [3, 4]. It is estimated, that there are almost 60 million people who are infected with HIV worldwide, of whom about 33.4 million living with HIV. Most of them are unaware that they are HIV carriers and may spread the virus to others [5].

Furthermore, unwanted pregnancy is one of the most common health problems with negative effects on individuals, families and society. It is estimated that approximately 60 % of all pregnancies are unplanned [6]. The most important reason of unintended pregnancy is that no contraception methods are used [7]. According to World Health Organization (WHO) in 2011, about 20 million unsafe abortions happened worldwide, almost all in developing countries [8].

Young people with low level of knowledge on sexual and reproductive health are more at risk of unintended pregnancies, unsafe abortions and sexually transmitted infections including HIV [3]. The most efficient way to avoid sexually transmitted infections is having sexual intercourse within a long-term and trusted monogamous partner and using condoms consistently and correctly [4].

In Malaysia the youth SRH is influenced by sexual relationship before marriage, sexually transmitted diseases (STD) including HIV/AIDS, unintended pregnancies and unsafe abortions [9, 10]. The Ministry of Health in Malaysia, in 2008, revealed that about 25 % of HIV infections are among people aged between 20 and 29 years [11]. An investigation has shown that the average age of the first sexual intercourse is 15 years and the percentage of premarital sexual activity has increased over the years in Malaysia [10]. Youths who initiated sexual intercourse early are less likely to use contraception and are at risk of unwanted pregnancy [12]. Data regarding the prevalence of abortion are not readily available due to their legal and moral ambiguities [13]. Recently there has been a growing number of reports in media on baby dumping in Malaysia, which has attracted a lot of attention and has posed a serious challenge to the country [14, 15].

Sexual and reproductive health (SRH) research in Malaysia has focused on different issues and fields of SRH among adolescences and adults. Research findings indicate a range of low mean scores of knowledge among school students [16, 17] and university students [18–20] to high mean scores of knowledge in female staff [21].

There are not many studies on sexual and reproductive health issues among Malaysian postgraduate students. Data on sexual and reproductive health knowledge among university students are important for planning effective educational programs in universities. The objective of this study was to determine the level of knowledge related to sexual and reproductive health among Malaysian postgraduate students in a public university, Malaysia.

## Methods

A cross-sectional study was conducted in a Malaysian university with 434 registered postgraduate students between September 5^th^, 2012 and September 15^th^, 2012. A systematic random sampling technique was used to select the sample. Data were collected at the School of Graduate Studies (SGS) from the list of postgraduate students during the registration timetable. Before the distribution of questionnaires, the purpose of the study was explained to them. The questionnaires took about 20 min to be completed and were collected immediately upon completion.

The data were collected using a self-administered pre-tested questionnaire in English which had been used in a study in Albania [22]. It consisted of questions related to socio-demographic factors and knowledge related to sexual and reproductive health, including STD knowledge, contraceptive knowledge and knowledge sources. Knowledge was determined using 47 questions on sexual and reproductive health with “Yes” or “No” or “Don’t Know” response.

The questionnaire was pre-tested on 40 students from a university other than the actual study participants’. After pre-testing of the questionnaire, the content validity was evaluated to examine each item for congruence. The standardized Cronbach’s alpha reliability coefficient was above 0.70. There were no modifications after pre-testing. This study was approved by the Ethical Committee of Universiti Putra Malaysia. A written consent was obtained from all the students before conducting the survey. All questionnaires were anonymous.

Respondents were given one point for answering correctly and zero for answering wrongly or not knowing the answer. Total knowledge scores can range between 0 and 47. Knowledge scores from 0 to 23 were considered as low knowledge while knowledge scores more than 23 was considered as having high knowledge regarding sexual health. The data were analyzed using Statistical Package for Social Sciences (SPSS) version 20.0. Comparisons among groups were made using appropriate inferential tests such as *t*-test and ANOVA. Multiple linear regression was used to determine the predictors of sexual and reproductive health knowledge. To estimate the proportion of variance in sexual and reproductive health knowledge that can be accounted for by socio-demographic factors and current educational status, multiple linear regression analysis was performed. The level of significance was set at 0.05.

### Sample size estimation

The number of postgraduate students needed for this study is calculated based on the previous study among university students in Malaysia [23]. The estimation sample size was estimated by the following formula [24].

$$ n=\frac{{\mathrm{Z}}_{1-\upalpha}\sqrt{2\mathrm{P}\left(1-\mathrm{P}\right)}+{\mathrm{Z}}_{1-\upbeta}\sqrt{{\mathrm{P}}_1\left(1-{\mathrm{P}}_1\right)+{\mathrm{P}}_2{\left(1-{\mathrm{P}}_2\right)}^2}}{{\left({\mathrm{P}}_1-{\mathrm{P}}_2\right)}^2} $$

Z_1-α_ = 1.96, Z_1-β_ = 1.282

P_1_ = 0.41 = Proportion of undergraduate students with poor attitude on sexual health

P_2_ = 0.58 = Proportion of undergraduate students with good attitude on sexual health

Total sample size with 20 % allowance for possible drop outs N = 434

## Results

### Socio-demographic characteristics of respondents

Out of the 434 respondents, the majority (78.6 %) were female and single (78.3 %). On average their age was 27 (±SD 4.305) ranging from 20 to 46 years. The mean age of the males and females were 26.82 (±SD 4.125) year and 27.41 (±SD 4.91) years, respectively. Majority of the respondents (70.2 %) were Malayfollowed by 18.2 % Chinese and 8 % Indian. More than half of the students were Muslims (73 %) (Table 1).

**Table 1:** Socio-demographic characteristics of respondents (n = 434)

Variable		Frequency	Percent
Gender	Male	93	21.4
	Female	341	78.6
Age (year)	<26 years	265	61.1
	≥26 years	169	38.9
Ethnicity	Malay	305	70.3
	Chinese	79	18.2
	Indian	35	8.1
	Other	15	3.5
Religion	Islam	317	73.0
	Christian	22	5.1
	Buddhist	58	13.4
	Hindu	33	7.6
	Other	4	0.9
Marital status	Married	94	21.7
	Single	340	78.3
Level of education	Master	369	85
	PhD	65	15
Faculty	Non- science	108	24.9
	Science	263	60.6
	Health science	63	14.5
Year of study	First year	349	80.4
	Others	85	19.6
Father’s education	Primary education	57	13.13
	Secondary education	239	55.07
	University degree	113	26.04
	Others	25	5.76
Mother’s education	Primary education	81	18.66
	Secondary education	266	61.29
	University degree	64	14.75
	Others	23	5.30

### Knowledge on sexual and reproductive health

The overall mean ± SD of sexual and reproductive knowledge score was 29.69 (±8.26). In this study, the majority (97.9 %) of the students knew AIDS is a sexually transmitted disease however, only a few (24.4 %) were aware of chlamydia. About 83.9 % perceived a person can acquire STD by receiving blood transfusion from an infected person and 73 % believed that a pregnant woman with STD/HIV can passthe disease to her unborn baby (Table 2).

**Table 2:** Sexually transmitted diseases knowledge (*n* = 434)

Item	Yes	No	Don’t know
1. Syphilis is a sexually transmitted disease.	353 (81.3)	7 (1.6)	74 (17.1)
2. Chlamydia is a sexually transmitted disease.	106 (24.4)	46 (10.6)	282 (65.0)
3. HIV/AIDS is a sexually transmitted disease.	425 (97.9)	3 (2.7)	6 (1.4)
4. Pneumonia is a sexually transmitted disease.	237 (54.6)	27 (6.2)	170 (39.2)
5. Hepatitis is a sexually transmitted disease.	188 (43.3)	106 (24.4)	140 (32.3)
6. Gonorrhea is a sexually transmitted disease.	161 (37.1)	57 (13.1)	216 (49.8)
7. Trichomonasis is a sexually transmitted disease.	51 (11.8)	52 (12.0)	331 (76.3)
8. Sexually transmitted infections can be transmitted by receiving blood transfusion from an infected person.	364 (83.9)	44 (10.1)	26 (6.0)
9. Sexually transmitted infections can be transmitted by using public toilets.	336 (77.4)	56 (12.9)	42 (9.7)
10. Sexually transmitted infections can be transmitted through kissing.	280 (64.5)	106 (24.4)	48 (11.1)
11. Sexually transmitted infections can be transmitted by unprotected sexual activity.	412 (94.9)	9 (2.1)	13 (3.0)
12. Sexually transmitted infections can be transmitted by unprotected intercourse.	349 (80.4)	26 (6.0)	59 (13.6)
13. Sexually transmitted infections can be transmitted through shaking hands.	400 (92.2)	7 (1.6)	27 (6.2)
14. Sexually transmitted infections can be transmitted from mother to unborn baby.	317 (73.0)	61 (14.1)	56 (12.9)
15. Using condom during sexual activity can always reduce the risk of getting STD.	384 (88.5)	22 (5.1)	28 (6.5)
16. Abstaining from sex can reduce the risk of getting STD.	90 (20.7)	251 (57.8)	93 (21.4)
17. Having only one partner can reduce the risk of getting STD.	372 (85.7)	31 (7.1)	31 (7.1)
18. Using withdrawal method can reduce the risk of getting STD.	143 (32.9)	109 (25.1)	182 (41.9)
19. Discharge from urethra or vagina is a symptom of STD.	310 (71.4)	25 (5.8)	99 (22.8)
20. Genital organs ulcer is a symptom of STD.	305 (70.3)	21 (4.8)	108 (24.9)
21. Vomiting is a symptom of STD.	184 (42.4)	58 (13.4)	192 (44.2)
22. Pain during sex is a symptom of STD.	255 (58.8)	55 (12.7)	124 (28.6)
23. Genital itching is a symptom of STD.	336 (77.4)	15 (3.5)	83 (19.1)
24. Headache is a symptom of STD.	197 (45.4)	62 (14.3)	175 (40.3)
25. Pain during urination is a symptom of STD.	265 (61.1)	50 (11.5)	119 (27.4)

The present study also showed that respondents had incorrect knowledge about STD prevention methods. More than half of the participants believed that the spread of sexually transmitted diseases was through public toilet (77.4 %), through kissing (64.5 %) and through shaking hands (92.1 %). However, most respondents believed that “use of condoms” (88.5 %), as well as “having one uninfected faithful sex partner” (85.7 %) can reduce the risk of sexually transmitted diseases. A few respondents (20.7 %) recognized abstinence from sex as a method of preventing sexually transmitted diseases.

In terms of the contraceptive methods knowledge, most of the respondents were aware of condoms (87.6 %), oral contraceptives (72.4 %), intrauterine device (IUD) (57.4 %), followed by morning after pill (45.4 %) and injectable contraception (49.1 %). In addition, a high percentage of the respondents (79.3 %) knew where to refer to when in need of medical treatment for sexual health issues.

This study shows that the most frequent sources of SRH information were internet (78.6 %) and newspaper (61.8 %). In contrast, parents (13.8 %) and friends (32.3 %) were the least frequent sources.

### Relationship between socio-demographic factors, current educational status and sexual and reproductive health knowledge

There were significant associations between age, marital status and faculty with sexual and reproductive health knowledge of the students (Table 3).

**Table 3:** Relationship between socio-demographic factors, current educational status and sexual and reproductive health knowledge (*n* = 434)

Variable	Group	N (%)	Mean score	Test	*p*-value
Gender	Male	93 (21.4)	30.989	t = 1.751	0.082
	Female	341 (78.6)	29.343		
Age (year)	<26 years	265 (61.1)	29.034	t = −2.167	0.031*
	≥26 years	169 (38.9)	30.733		
Ethnic	Malay	305 (70.3)	29.459	t = −0.899	0.37
	Non- Malay	129 (29.7)	30.255		
Religion	Muslim	317 (73)	29.425	t = −1.118	0.265
	Non- Muslim	117 (27)	30.427		
Marital status	Married	94 (21.7)	31.904	t = 3.26	0.001*
	Single	340 (78.3)	29.085		
Year of study	First year	349 (80.4)	29.352	t = −1.842	0.068
	Others	85 (19.6)	31.105		
Faculty	Non- science	108 (24.9)	26.546		0.000*
	Science	263 (60.6)	29.4981	F = 29.103	
	Health science	63 (14.5)	35.9206		

*Level of significance (*p* < 0.05)

### Sexual and reproductive health knowledge and associated factors

A standard multiple regression was conducted to estimate the prediction of knowledge by socio-demographic factors and current educational status. Multiple linear regression analysis showed that sexual and reproductive health knowledge were associated with marital status and faculty of the students. However age, gender, ethnicity, religion and year of study were not associated with sexual and reproductive health knowledge. The assumptions of normality, linearity and homoscedasticity of residuals were met. In combination, socio-demographic factors and current educational status accounted for a significant 9 % of the variability in sexual and reproductive health knowledge, R^2^ = 0.09, adjusted R^2^ = 0.074, *f* (7, 426) = 11, *p* <0.001 (Table 4).

**Table 4:** Multiple linear regression analysis showing factors associated with sexual and reproductive health knowledge

Models	Unstandardized coefficients β	Std. error	t	Sig.	95 % confidence interval for β	
(Constant)	46.354	4.671	9.924	.000	37.173	55.535
Marital status	−3.507	1.095	−3.203	.001*	−5.659	−1.355
Faculty	−4.655	.620	−7.50	.000*	−5.875	−3.436
Gender	−.948	.905	−1.047	.296	−2.727	.831
Age	.040	.105	.381	.704	−.167	.247
Ethnic	−.733	.662	−1.10	.268	−2.033	.567
Religion	329	.502	.655	.513	−.658	1.315
Year of study	.417	.567	.736	.462	−.697	1.531

*Level of significance (*p* <0.05)

## Discussion

In this study, we showed that the level of sexual and reproductive health knowledge was low among postgraduate students. The lack of knowledge on sexual and reproductive health was reported in most of the developing countries [25]. Inadequate sexual health knowledge among youths may lead to risky behaviors, unsafe sexual practices, sexually transmitted diseases and unwanted pregnancies [26, 27]. This finding is consistent with a study by Wong et al. [28] regarding sexual and reproductive health among university students in Malaysia [26]. Conversely, Simbar et al. [29] found greater levels of sexual and reproductive health knowledge among university students in Iran. This may be due to steady educational programs in Iranian university students [29]. There is evidence showing that sex education result in preventing high risk sexual behavior and it ia not associated with increasing sexual activity [30].

The present study showed that the contraceptive methods knowledge was also low in most of the postgraduate students. Our results are consistent with the finding of the other study among Lebanese university students [31]. In contrast, knowledge of contraceptive issues was reported in high level among university students in Greece [32] and Iran [29]. Recently all Iranian college students are educated about family planning at university, it seems that this cause to increase level of contraceptive knowledge among university students. These efforts should be adapted to their gender and their field of study, with improved educational program for non-medical students [29]. It seems that an educational program on contraception is also necessary for university students [29]. It has been shown that sex education that includes contraception does not increase sexual activity, but rather persuades correct and consistent use of contraception for preventing unwanted pregnancy and sexually transmitted diseases [30, 33].

The results of this study showed that many sources of information about sexual and reproductive health are available for university students. Despite these efforts, however, the lack of accurate knowledge and misconceptions about sexually transmitted diseases were seen. As the results indicated, over half of the students believed that sexually transmitted diseases were spread by using public toilet, through kissing and shaking hands. It seems they do not have enough knowledge about transmission of sexual diseases. Furthermore, there are other factors that may affect the level of sexual knowledge, such as religious values and cultural sensitivities. Topics on sexual issues are still considered a taboo in this society and students are not exposed to such matters [28]. Similar misconceptions regarding prevention of sexually diseases were observed among university students in two previous studies in Malaysia [23, 28]. These findings may also suggest that there is insufficient education and coverage of such content by the mass media and educational institutions [23].

The current study showed that married and older students had higher mean knowledge score compare with younger students. As individuals grow older, their sexual curiosity and development lead them to seek for more information relating to sexual issues. These findings were consistent with those of other similar studies on Malaysian university students [20, 23]. Furthermore, the results of the present study showed students in health science faculties had higher sexual and reproductive knowledge than other students. This fact may be somewhat due to the university courses that cover subjects on sexual and reproductive health. These results were supported by a study among Iranian university students [29]. Furthermore, an educational program on sexual and reproductive health is necessary for non-science students.

In the present research, the most frequently referred sources of sexual and reproductive health information were the internet and newspaper while the least frequent sources were parents and friends. These results are in line with those of two similar studies in Turkey and Malaysia among university students [34, 28]. In contrast a study of Chinese university students showed that the majority of the students gained sexual information by magazines and friends [35]. Anticipatory, parents are important source of information on sexual matters; but that was not recognized by most students as a source of information [36]. This finding is consistent with the findings of Anwar et al. (2010) on awareness of school students related to sexually transmitted infections in Pulau Pinang, Malaysia, in which it was found families were the most uncommon sources of information on sexual matters [36]. The previous study in Malaysia revealed that students were less likely to communicate with parents about sexual issues because of the fear that their parents think they had engaged in sexual activity [28].

The use of self-administered questionnaires may be considered a limitation of the present study. Sexual issues are sensitive topics regarded taboos by many youths who are reluctant to talk aboutthem. This can also be a limitation of this study. A socially-desirable response bias and an information bias were considered in this study.

## Conclusions

In conclusion, the finding of the current study showed that the level of sexual and reproductive health knowledge is not satisfactory and there are still some misconceptions about sexual issues. Inadequate knowledge may place students at risk of sexually transmitted infections including HIV/AIDS and unwanted pregnancy. Sexual and reproductive health knowledge was associated with marital status and faculty among students. Thus, educational programs in the form of short courses or peer education can improve the sexual and reproductive health knowledge among university students. In some studies, peer education has brought positive changes in all aspect of knowledge, attitude and practice [37].

## Abbreviations

AIDS: Acquired immune deficiency syndrome
CDC: Centers for disease control and prevention
MLR: Multiple linear regression
HIV: Human immunodeficiency virus
SRH: Sexual and reproductive health
STD: Sexually transmitted disease
UPM: Universiti Putra Malaysia
UNAIDS: United Nation Joint Program on HIV/AIDS
UNGASS: United Nations General Assembly Special Session on HIV/AIDS
WHO: The World Health Organization
